# Supplementary material for: Influence of teaching a structured and humanized method of care on the perception of medical student attitudes in the doctor-patient relationship
Source: PLoS One. 2025 Feb 7;20(2):e0314317. doi: 10.1371/journal.pone.0314317 (PMC11805414; doi:10.1371/journal.pone.0314317)
Supplement: S1 File — (DOCX) [file pone.0314317.s001.docx]

*Supporting Information:*

*PATIENT-PRACTITIONER ORIENTATION SCALE (PPOS)*

*
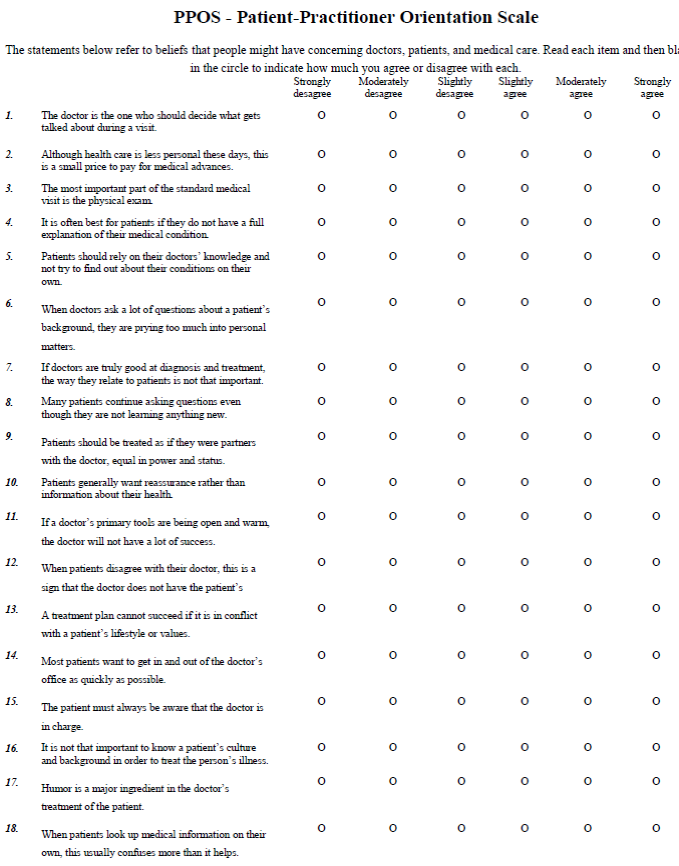
*

*Permission from the owner of the original questionnaire to use it:*

*
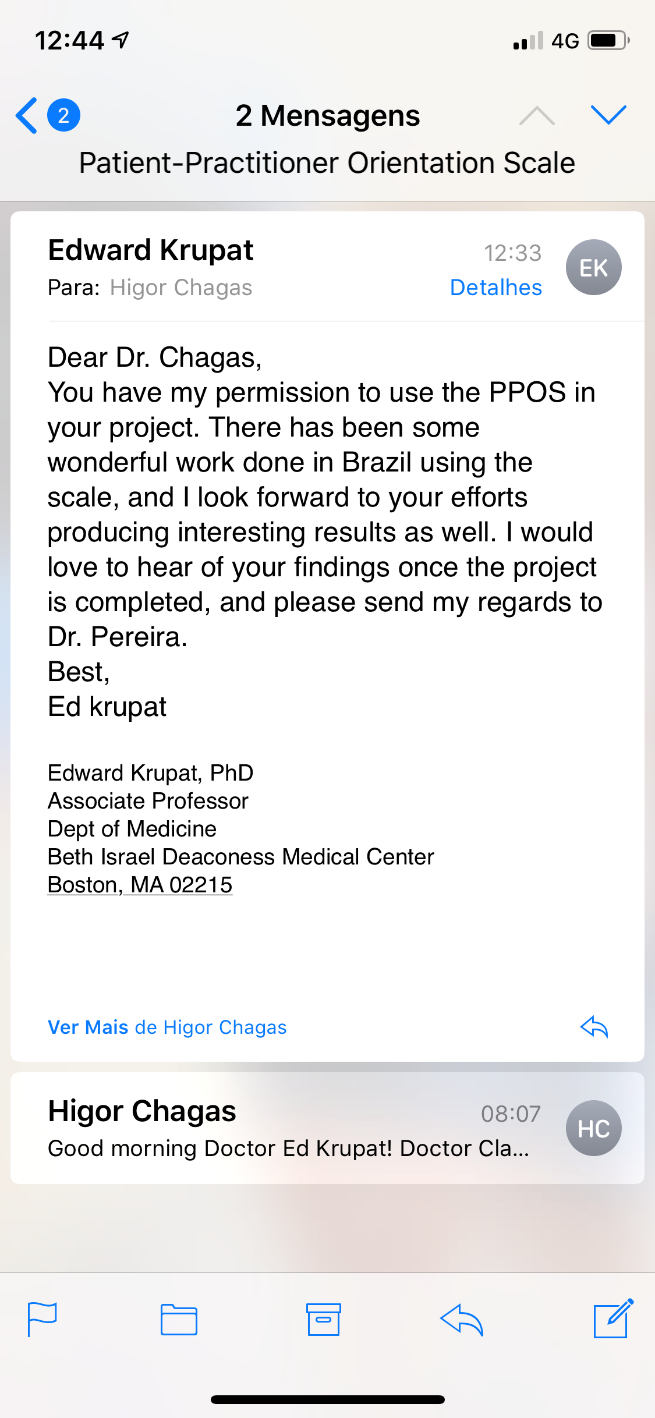
*

*PATIENT-PRACTITIONER ORIENTATION SCALE (PPOS)* translated and validated for Portuguese in Brazil:

*ESCALA DE ORIENTAÇÃO MÉDICO-PACIENTE (EOMP)*

*
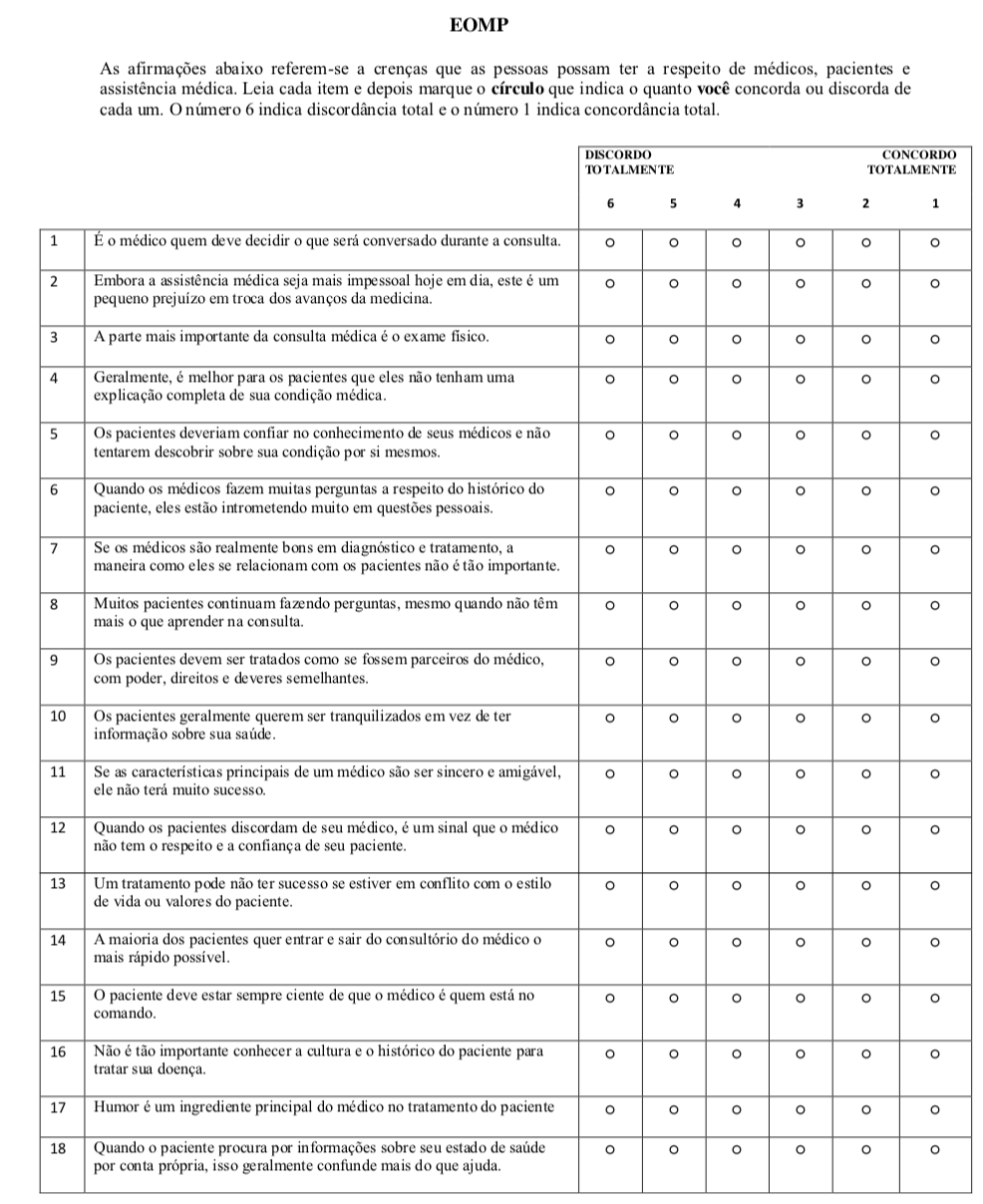
*
